# Changes in the minor salivary glands and ductal carcinoma resulting from induction of oral carcinogenesis

**DOI:** 10.1007/s10735-026-10938-5

**Published:** 2026-08-26

**Authors:** Caio Rodrigues Maia, Raissa Cláudia Eufrázio de Oliveira, Viviane Tainá Carvalho da Silva, Cecília Cerqueira Souza, Vivian Gracielly Nascimento de Oliveira, Alana Beatriz Brito Costa, Pedro Paulo de Andrade Santos

**Affiliations:** 1 Federal University of Rio Grande do Norte (UFRN), Natal, RN Brazil; 2 Department of Morphology – Biosciences Center, Federal University of Rio Grande do Norte (UFRN), Campus Universitário, Lagoa Nova, Natal, RN 59078-900 Brasil

**Keywords:** 4NQO, Palatine salivary glands, Tongue salivary glands, Ductal carcinoma, Squamous cell carcinoma

## Abstract

Given the scarcity of experimental studies on the effects of induced oral carcinogenesis on the minor salivary glands, the aim of this study was to analyze the histopathological changes present in the lingual and palatal minor salivary glands (MiSGs) resulting from 4NQO exposure in rats from the 8th to the 30th week of the experiment. Eleven rats were used in the control group and 45 rats in the experimental groups that received 4NQO/propylene glycol diluted in drinking water. Histopathological analysis revealed evidence of hyperplasia, metaplasia, and ductal dysplasia, saliva buildup in the ducts, inflammatory infiltrate, lobular atrophy, degeneration of the secretory portion, fibrosis/replacement, and invasion of the minor salivary glands. The most evident change in both tongue and palate MiSGs was degeneration of the secretory portion, which reduced salivation and promoted the oncogenic action of 4NQO. However, changes such as hyperplasia, metaplasia, and dysplasia, which may promote the development of neoplastic changes, were more prevalent in tongue MiSGs. In the present study, we also identified an incidental case of salivary ductal carcinoma in palatal MiSGs. Given this, it is important to be vigilant regarding potential changes in MiSGs, as they may be related to the development of malignancies.

## Introduction

The minor salivary glands (MiSGs) are very numerous and are widely distributed throughout the oral cavity, with the exception of the gingiva, the anterior third of the hard palate, and the anterodorsal region of the tongue. The MiSGs are located in the submucosa and are surrounded by connective tissue or muscle tissue (Aframian et al. 2019). Although they account for less than 10% of total daily saliva secretion, the MiSGs maintain a continuous flow of saliva with a high concentration of protective substances such as mucins and immunoglobulin A (de Moraes et al. 2025).

The most common cause of oral cancer is the use of tobacco and related products. These chemical carcinogens cause damage to the nuclei of keratinocytes, not only in the oral lining epithelium but also in the epithelium of the excretory ducts, potentially leading to epithelial dysplasia and squamous cell carcinoma in the salivary glands as well (Mohan et al. 2016).

It is known that 4NQO (4-nitroquinoline-1-oxide) is a synthetic carcinogen responsible for causing systemic and molecular changes; it is widely used and capable of reproducing the evolutionary stages of oral carcinogenesis, ranging from epithelial dysplasia to invasive squamous cell carcinoma (Das et al. 2024; Sahu et al. 2023).

In the present study, we chemically induced oral lesions across the spectrum of malignancy in Wistar rats over a maximum experimental period of 30 weeks. This was followed by a histopathological analysis of the changes present in the lingual and palatal MiSGs. We also report evidence of an incidental case of salivary ductal carcinoma possibly related to experimental induction.

## Materials and methods

### Animals and Ethics

The present animal experiment complied with the ARRIVE guidelines and carried out in accordance with the U.K. Animals (Scientific Procedures) Act, 1986 and associated guidelines, EU Directive 2010/63/EU for animal experiments, or the National Institutes of Health guide for the care and use of Laboratory animals (NIH Publications No. 8023, revised 1978). This experiment was conducted with 56 male albino Wistar rats, starting at 10 weeks of age and weighing between 350 and 400 g. This study was approved by the Ethics Committee on Animal Use (CEUA/UFRN) under No. 051/2022.

### Monitoring of animals during the experiment

The rats were housed in a vivarium with specific conditions, free of pathogens and under veterinary supervision. They were kept in ventilated cages (maximum of five rats per cage) with environmental enrichment, undergoing a one-week adaptation period before the start of the study. During the study period, the rats were kept at controlled temperature and humidity, under a 12-hour light/dark cycle, with ad libitum access to food and water. All rats were weighed at the beginning of the experiment, with a new weighing performed every seven days, and the last weighing performed before euthanasia. Of the 45 experimental rats, we lost one around the 23rd week; the animal was found dead many hours after the incident. Consequently, we excluded this rat from our sample, resulting in a total of 44 experimental rats analyzed.

### Protocol and euthanasia

**Fig. 1:**
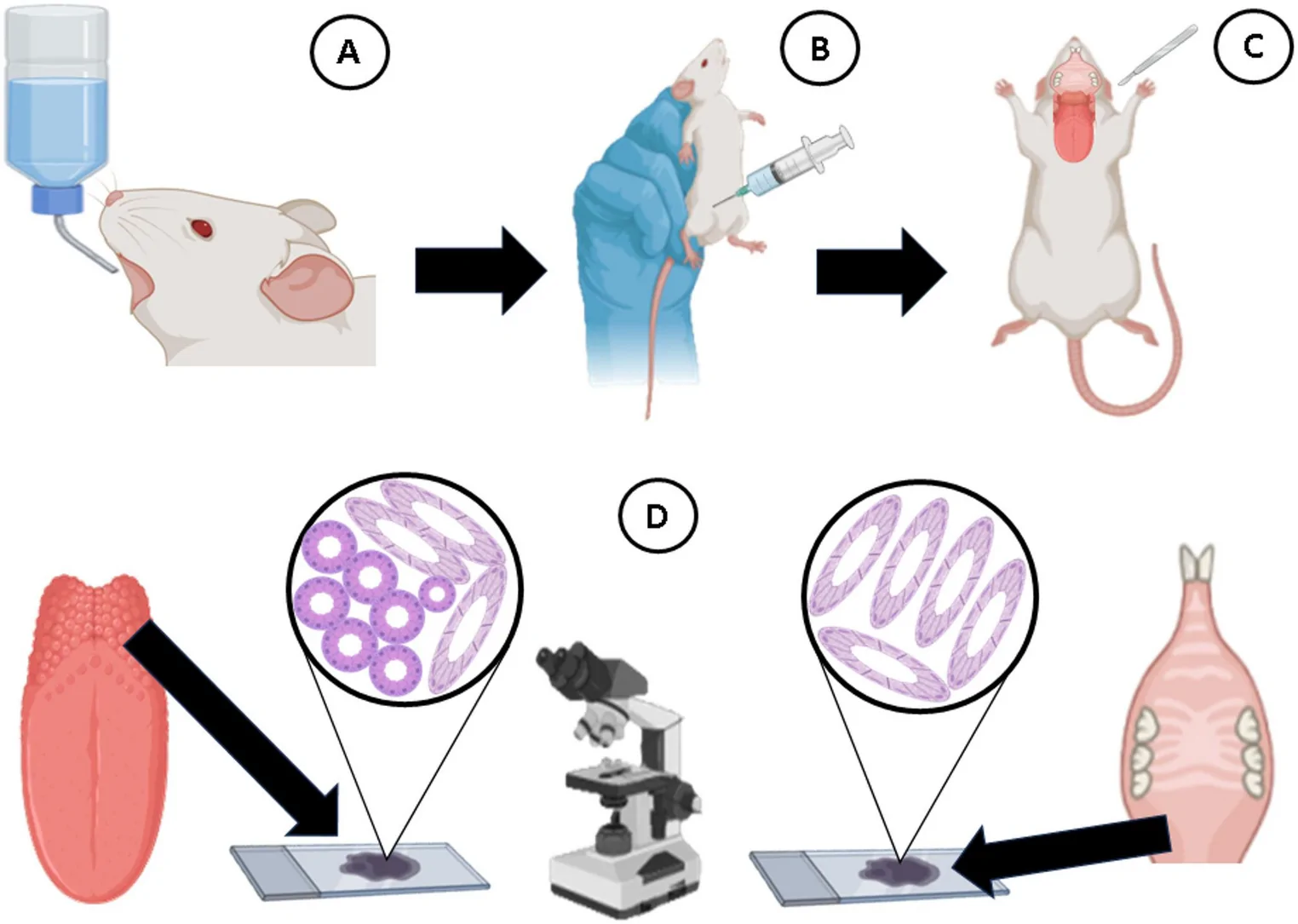
**A** Hydration with drinking water + 4NQO/propylene glycol and ad libitum feeding; **B** Euthanasia via intraperitoneal injection; **C** Rat positioned in dorsal recumbency for removal of tongue and the entire palate (hard and soft); **D** Laboratory processing with histological slides obtained for microscopic analysis and histopathological analysis of minor salivary glands. Created with BioRender.com

For the development of oral carcinogenesis (Fig. 1A–D), we conducted the experiment with 11 control rats and 44 experimental rats that used 4NQO (Sigma-Aldrich). The stock solution of 4NQO/Propylene Glycol (5 mg/ml) at − 20 °C (Sagheer et al. 2021) was diluted in the rats’ drinking water, obtaining a final solution of 20 mg per liter of water (20 ppm). The final solution consisting of 4NQO/Propylene Glycol/Fresh Water was replaced once a week. Any leftovers from the final solution were stored in a specific container for the disposal of toxic waste.

**Fig. 2:**
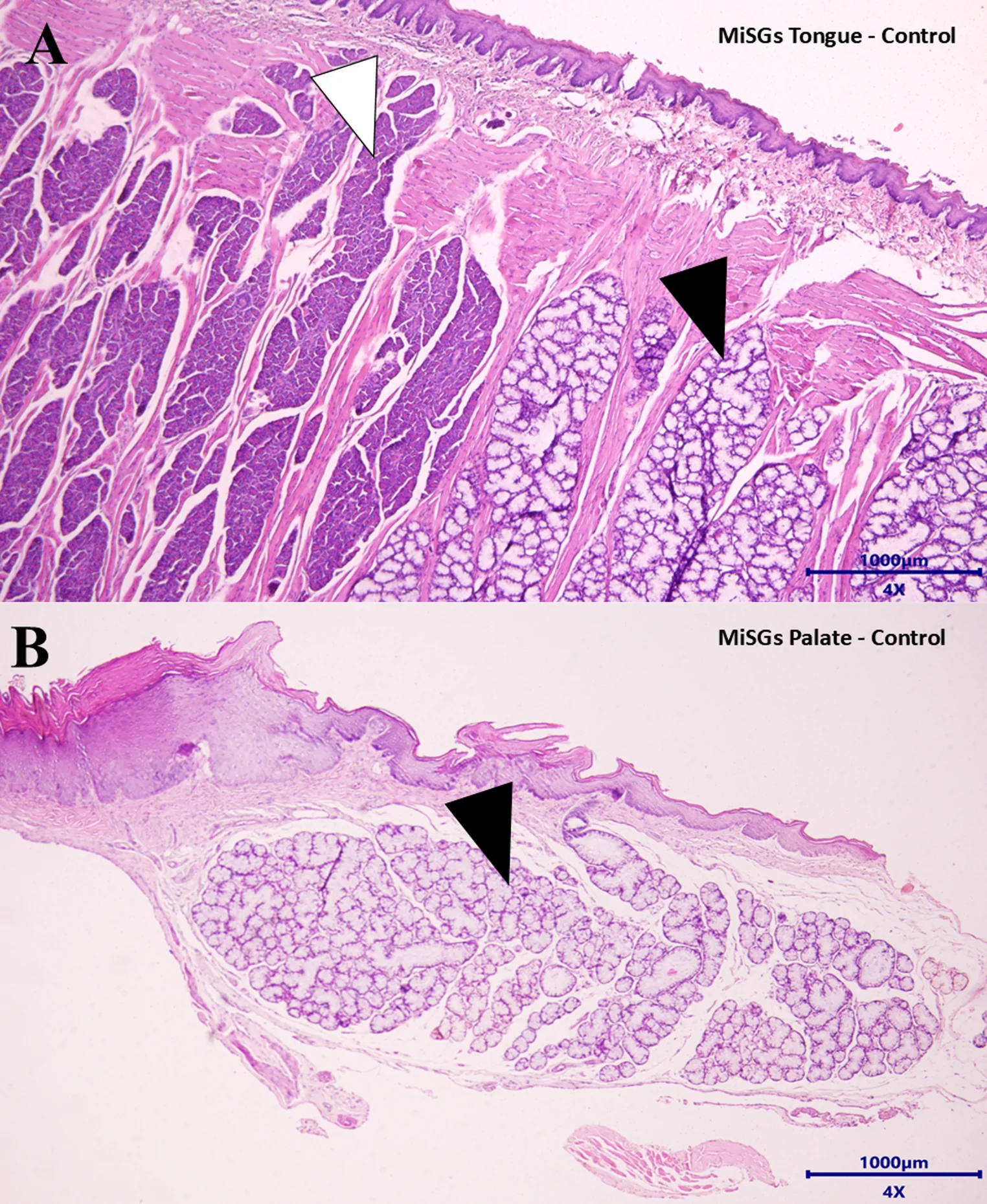
**A** Photomicrograph showing the control group of minor salivary glands (white arrow) serous acinar (Von Ebner) and mucous tubular (black arrow) in the tongue (HE, 4x objective); **B** Photomicrograph showing the control group of mucous tubular minor salivary glands in the palate (HE, 4x objective);

To euthanize the rats that presented lesions in the palate, the animals belonging to the experimental and control groups were weighed beforehand. After that, a lethal dose of 5% Ketamine Hydrochloride + 2% Xylazine (intraperitoneal) was administered. The criteria for humane euthanasia were body weight loss > 20% and signs of decreased animal welfare, including physical appearance, behavior, and respiratory distress. Upon confirming the absence of reflexes when pinching the rats’ tails, the surgical procedure to remove the entire hard and soft palate was performed. The collected material was immediately immersed in a 10% formaldehyde solution for subsequent laboratory processing and preparation of slides for histopathological analysis.

### Histological processing

Hematoxylin-eosin (HE) staining produces blue nuclear staining and pink cytoplasmic staining (Tolosa et al. 2003). The sections were deparaffinized with xylene and rehydrated using graded alcohols to distilled water, then stained with Harris hematoxylin for 2 min. After washing with running water, the sections were immersed in a saturated lithium carbonate solution to accelerate the oxidation of hematoxylin and allow the nuclei to stain blue. Subsequently, another wash with running water was performed, and staining with alcoholic eosin was carried out for 4 min. Finally, the sections were dehydrated, cleared, mounted, and covered with coverslips (Rodrigues et al. 2021).

### Histopathological analysis

All slides obtained were analyzed under an optical microscope (OPTIKA B-800, Italy) and photographed using the image capture system (OPTIKA PROVIEW - OPTIKAM CP6, Italy). The study examined the rats in the control group (Fig. 2A, B) and the experimental group, investigating the presence of hyperplasia, metaplasia, and ductal dysplasia; saliva build up in the duct; inflammatory infiltrate; lobular atrophy; degeneration of the secretory portion; fibrosis/replacement; and invasion of the minor salivary glands (MiSGs) of the tongue and palate were categorized as 0 (absent) and 1 (present). Each experimental induction week evaluated received the following ordinal categorization: 1 (8 weeks); 2 (12 weeks); 3 (16 weeks); 4 (19 weeks); 5 (20 weeks); 6 (22 weeks); 7 (23 weeks); 8 (24 weeks); 9 (25 weeks); 10 (26 weeks); 11 (27 weeks); 12 (28 weeks); 13 (30 weeks).

Each case was analyzed by 2 pre-calibrated examiners under light microscopy. The calibration was carried out in two stages: The first stage consisted of preselecting the histological features to be studied and defining them, as well as holding preliminary meetings with the research group. The second stage involved assessing the level of agreement and resolving discrepancies in the histopathological evaluation.

### Immunohistochemistry

Three-micrometer section sample were prepared from paraffin-embedded tissue block of the case of salivary duct carcinoma. The tissue sections were deparaffinized and immersed in 3% hydrogen peroxide to block endogenous peroxidase activity. The sections were then washed in phosphate-buffered saline (PBS). The following primary antibodies were employed: Mouse Anti-Human Gata-3 Monoclonal antibody (1:25; B10; sc-137152; Santa Cruz Biotechnology Inc. Dallas, USA). After treatment with normal serum, the sample sections were incubated with the primary antibodies in a moist chamber followed by washing twice with a 1% Tween 20 solution prepared in TRIS–HCl (pH 7.4). They were then treated at room temperature employing an HRP polymer HiDef detection system (Cell Marque, California, USA) for anti-Gata-3. Peroxidase activity was verified by immersing the tissue sections in diaminobenzidine (Liquid DAB + Substrate; Dako), resulting in a brown reaction product. Finally, the sections were counterstained with Harris’ hematoxylin and cover slipped. Gata-3 (B10) was routinely validated by a western blot (WB) analysis according to the manufacturer’s specifications (Santa Cruz) and analyzed in Jurkat (A), CCRF-HSB-2 (B), and Molt-4 (C) whole cell lysates, where the antibody labeled a band of about 50 kDa.

### Statistical analysis

The data from the histopathological analysis were subjected to statistical analysis using the Statistical Package for the Social Sciences (SPSS) software (version 20.0; SPSS Inc.; Chicago, IL, USA). After applying the Kolmogorov-Smirnov normality test, we found that the data were nonparametric. Therefore, we performed the chi-square (χ^2^) test, followed by Spearman’s correlation test (*r*_*s*_), with a significance level of 5% (*p* < 0.05).

## Results

**Fig. 3:**
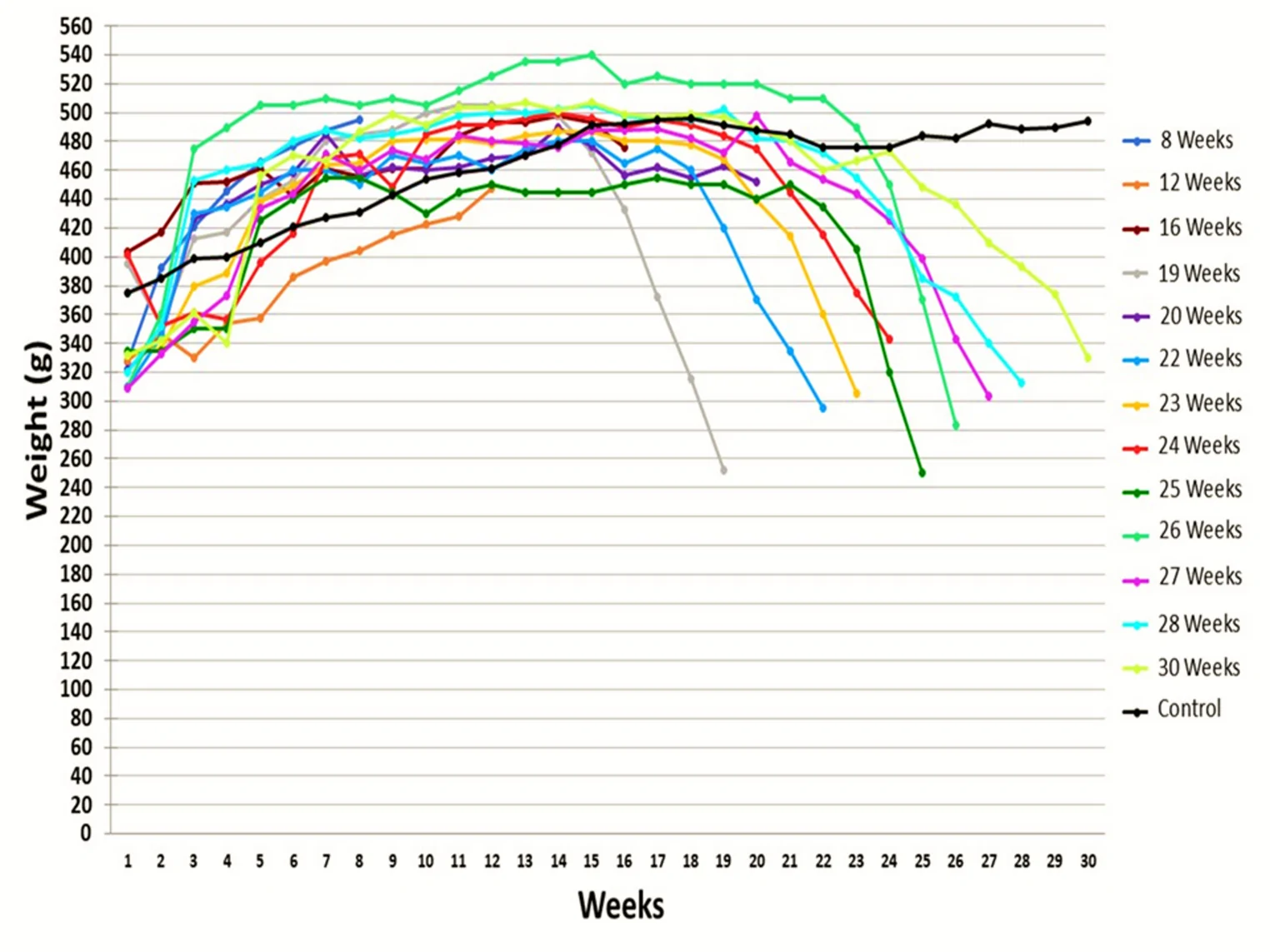
Line graphs showing the measurement of the weights of rats in the experimental groups (8th to 30th week) and control group

The weight monitoring of rats (Fig. 3) in the experimental groups aged 8 to 16 weeks presented a clinical picture within expectations, without greater severity. From the 19th experimental week onwards, we observed a considerable variation in the clinical picture of the animals, with a consequent increase in the number of animals requiring euthanasia in the 23-week group. As previously established in our study, scheduled euthanasia was performed every 4 weeks starting in the 8th week after induction with 5 animals euthanized per experimental week. However, this was only possible until the 16th experimental week, at which point, due to the fulfillment of the humanitarian criteria for euthanasia, we had to bring forward some euthanasia procedures, leading to an irregularity in the number of rats euthanized per experimental week. Even with this issue, we were able to reach the 30th experimental week with 3 rats (Table 1).

**Table 1:** Flowchart showing the monitoring of the mice throughout the experimental weeks

Experimental week	Number of animals evaluated	Number of animals euthanized	Specific reason for euthanasia	Category/justification
1 to 7 weeks	56	0	–	–
8 weeks	56	5	Euthanasia by appointment	End of the trial period
9 to 11 weeks	51	0	–	–
12 weeks	51	5	Euthanasia by appointment	End of the trial period
13 to 15 weeks	46	0	–	–
16 weeks	46	5	Euthanasia by appointment	End of the trial period
17 to 18 weeks	41	0	–	–
19 weeks	41	2	Severe shortness of breath; Body weight loss > 20%.	Humanitarian euthanasia (relief from severe suffering)
20 weeks	39	3	Euthanasia by appointment	End of the trial period
21 weeks	36	0	–	–
22 weeks	35	1	Severe shortness of breath; Body weight loss > 20%.	Humanitarian euthanasia (relief from severe suffering)
23 weeks	34	7	Severe shortness of breath; High pain scale; Body weight loss > 20%.	Humanitarian euthanasia (relief from severe suffering)
		1	Found dead	Excluded from the sample
24 weeks	26	4	Euthanasia by appointment	End of the trial period
25 weeks	22	1	Severe shortness of breath; High pain scale; Body weight loss > 20%.	Humanitarian euthanasia (relief from severe suffering)
26 weeks	21	1	Severe shortness of breath; Body weight loss > 20%.	Humanitarian euthanasia (relief from severe suffering)
27 weeks	20	5	Severe shortness of breath; High pain scale; Body weight loss > 20%.	Humanitarian euthanasia (relief from severe suffering)
28 weeks	15	2	Euthanasia by appointment	End of the trial period
29 weeks	13	0	–	–
30 weeks	13	13 (3 experimental and 11 control)	Euthanasia by appointment	End of the trial period

**Fig. 4:**
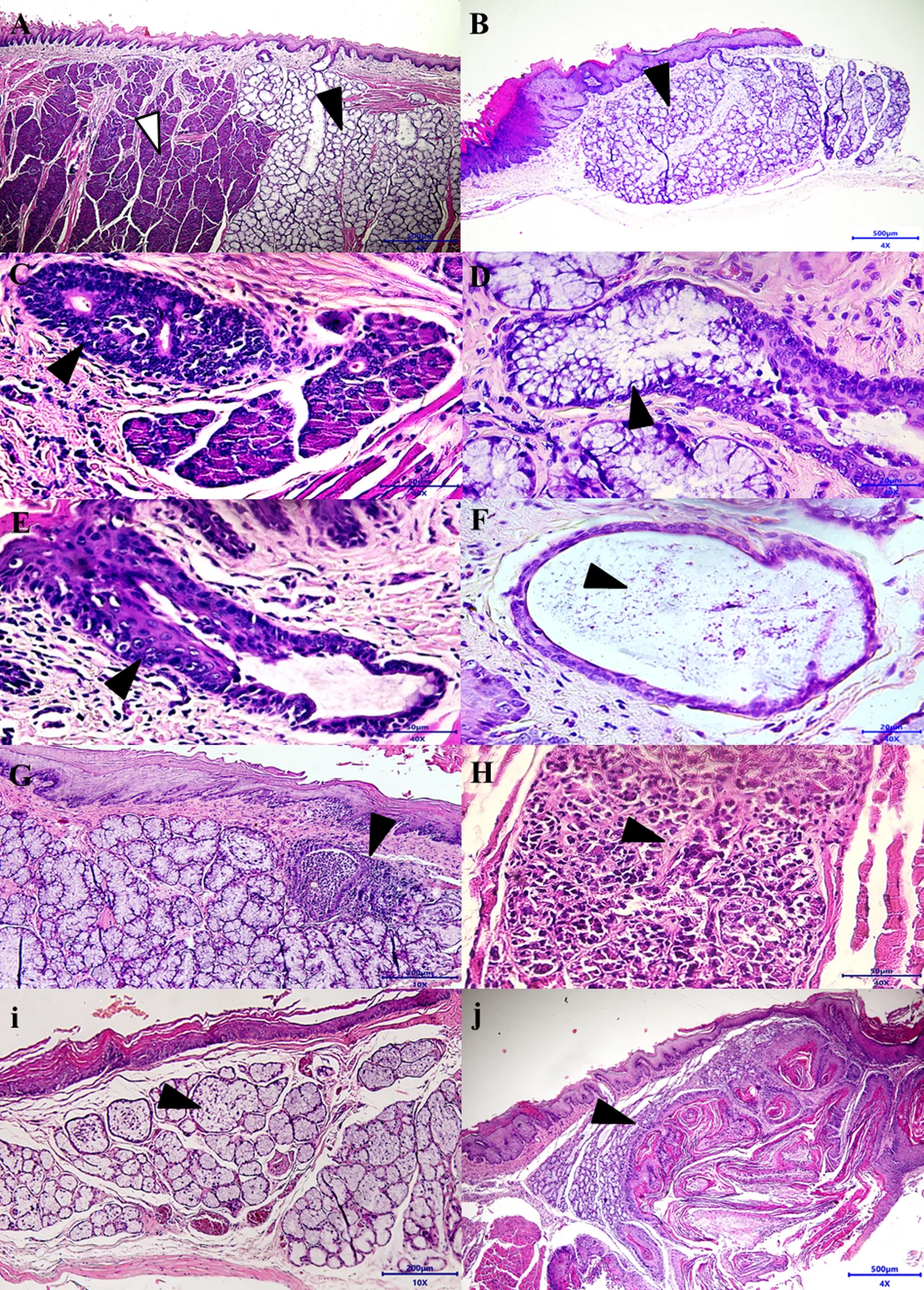
**A** Photomicrograph showing the experimental group of minor salivary glands (white arrow) serous acinar (Von Ebner) and mucous tubular (black arrow) in the tongue (HE, 4x objective); **B** Photomicrograph showing the experimental group of mucous tubular minor salivary glands in the palate (HE, 4x objective); **C** Ductal epithelial hyperplasia with intraductal proliferation and bridging between ductal epithelial cells (HE, 40x objective); **D** Mucosal metaplasia of the ductal epithelium (HE, 40x objective); **E** Ductal epithelial dysplasia (HE, 40x objective); **F** Accumulation of mucin in an ectatic duct (HE, 40x objective); **G** Inflammatory infiltrate in the glandular parenchyma (HE, 10x objective); **H** Fibrosis and lobular atrophy (HE, 40x objective); **I** Degeneration of the secretory portion (HE, 10x objective); **J** Invasion of the minor salivary gland by squamous cell carcinoma (HE, 4x objective)

**Fig. 5:**
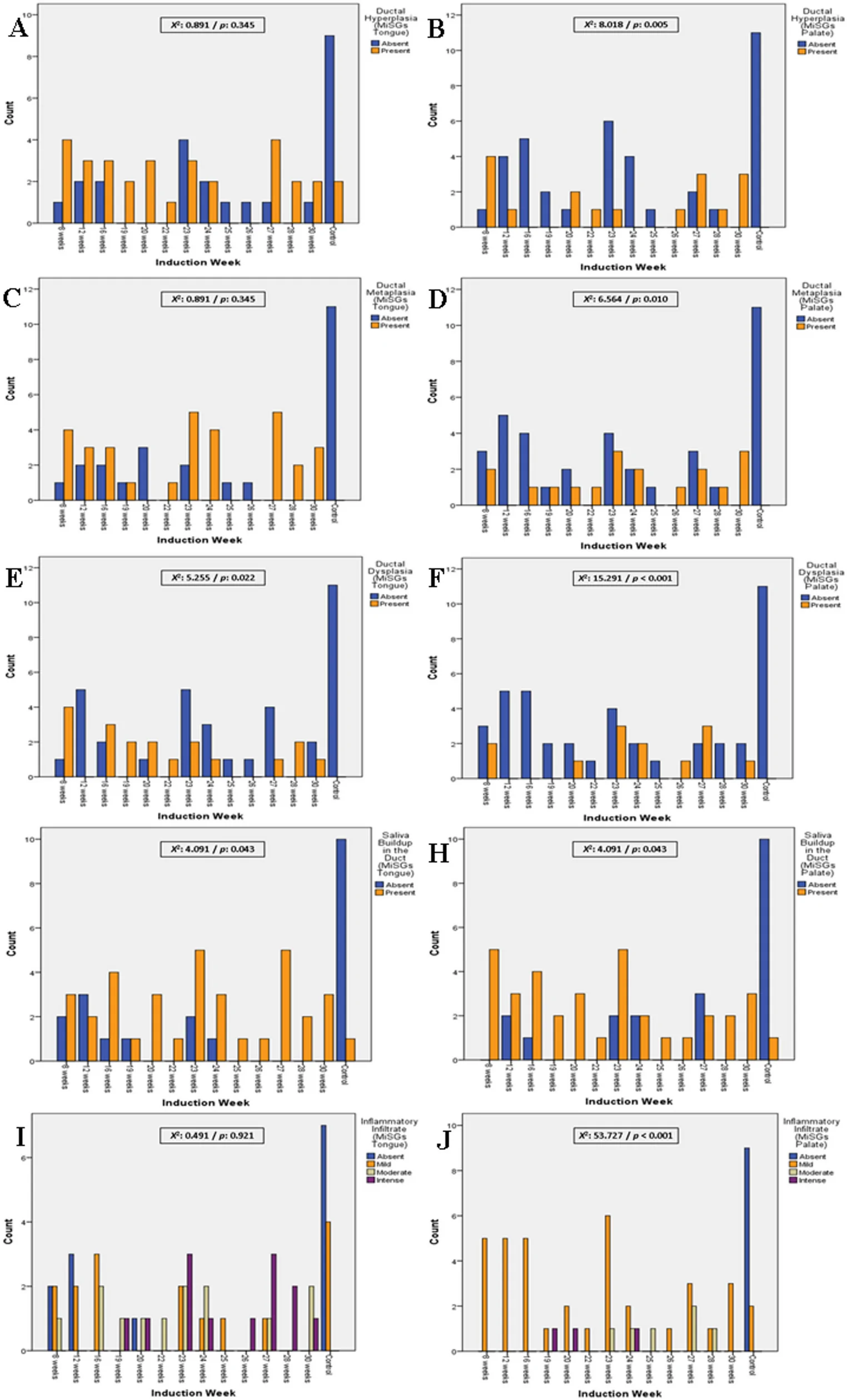
Bar charts showing the histopathological features observed in the minor salivary glands (MiSGs): **A** Ductal hyperplasia in the MiSGs of the tongue; **B** Ductal hyperplasia in the MiSGs of the palate; **C** Ductal metaplasia in the MiSGs of the tongue; **D** Ductal metaplasia in the MiSGs of the palate; **E** Ductal dysplasia in the MiSGs of the tongue; **F** Ductal dysplasia in the MiSGs of the palate; **G** Accumulation of saliva within the duct in the MiSGs of the tongue; **H** Accumulation of saliva within the duct in the MiSGs of the palate; **I** Inflammatory infiltrate in the MiSGs of the tongue; **J** Inflammatory infiltrate in the MiSGs of the palate

Considering the histopathological changes (Fig. 4A–J) observed in the minor salivary glands (MiSGs) of the tongue (*n* = 44) and palate (n: 44) of rats subjected to chemical induction of oral carcinogenesis, we found that over the weeks of experimental induction, there was a predominance of approximately 66% of ductal epithelial hyperplasia (Fig. 5A, B) in the MiSGs of the tongue (n: 29), compared to 38.6% (n: 17) in the MiSGs of the palate (Table 2). Ductal epithelial metaplasia (Fig. 5C, D) was also more evident in approximately 70% of tongue MiSGs (n: 31) compared to palatal MiSGs, which had 18 cases. As for ductal dysplasias (Fig. 5E, F), they were found in 19 cases (43%) of tongue MiSGs and 13 cases (29.5%) of palate MiSGs. Saliva accumulation within the duct and consequent ductal ectasia (Fig. 5G, H) were present in 34 cases (77%) in both tongue and palate MiSGs. In the analysis of the inflammatory infiltrate, we observed in lingual MiSGs an intense inflammatory infiltrate (Fig. 5I) in 13 cases (29.5%), moderate in 13 cases (29.5%), mild in 12 cases (27%), and absent in 6 cases (14%). In contrast, in palatal MiSGs (Fig. 5J), a mild inflammatory infiltrate was predominant in 35 cases (79.5%), with 6 cases (13.7%) showing moderate and 3 (6.8%) showing intense infiltrate.

**Table 2:** Changes observed in the MiSGs of the tongue and palate following experimental induction of oral carcinogenesis in absolute values (n) and percentages (%)

Changes in MiSGs	Tongue MiSGs (*n*: 44)		Palate MiSGs (*n*: 44)		Total MiSGs (*n*: 88)	
	*n*	%	*n*	%	*n*	%
Ductal hyperplasia	29	65.9	17	38.3	46	52.3
Ductal metaplasia	31	70.5	18	40.9	49	55.7
Ductal dysplasia	19	43.2	13	29.5	32	36.4
Saliva buildup in the duct	34	77.3	34	77.3	68	77.2
Infiltrate inflammatory	38	86.4	44	100	82	93.1
Lobular atrophy	39	88.6	14	31.8	53	60.2
Degeneration of the secretion portion	43	97.7	43	97.7	86	97.7
Fibrosis/other glandular replacement	40	91	22	50	62	70.4
Glandular invasion	24	54.5	6	13.6	30	34.1

MISGs: Minor Salivary Glands.

**Fig. 6:**
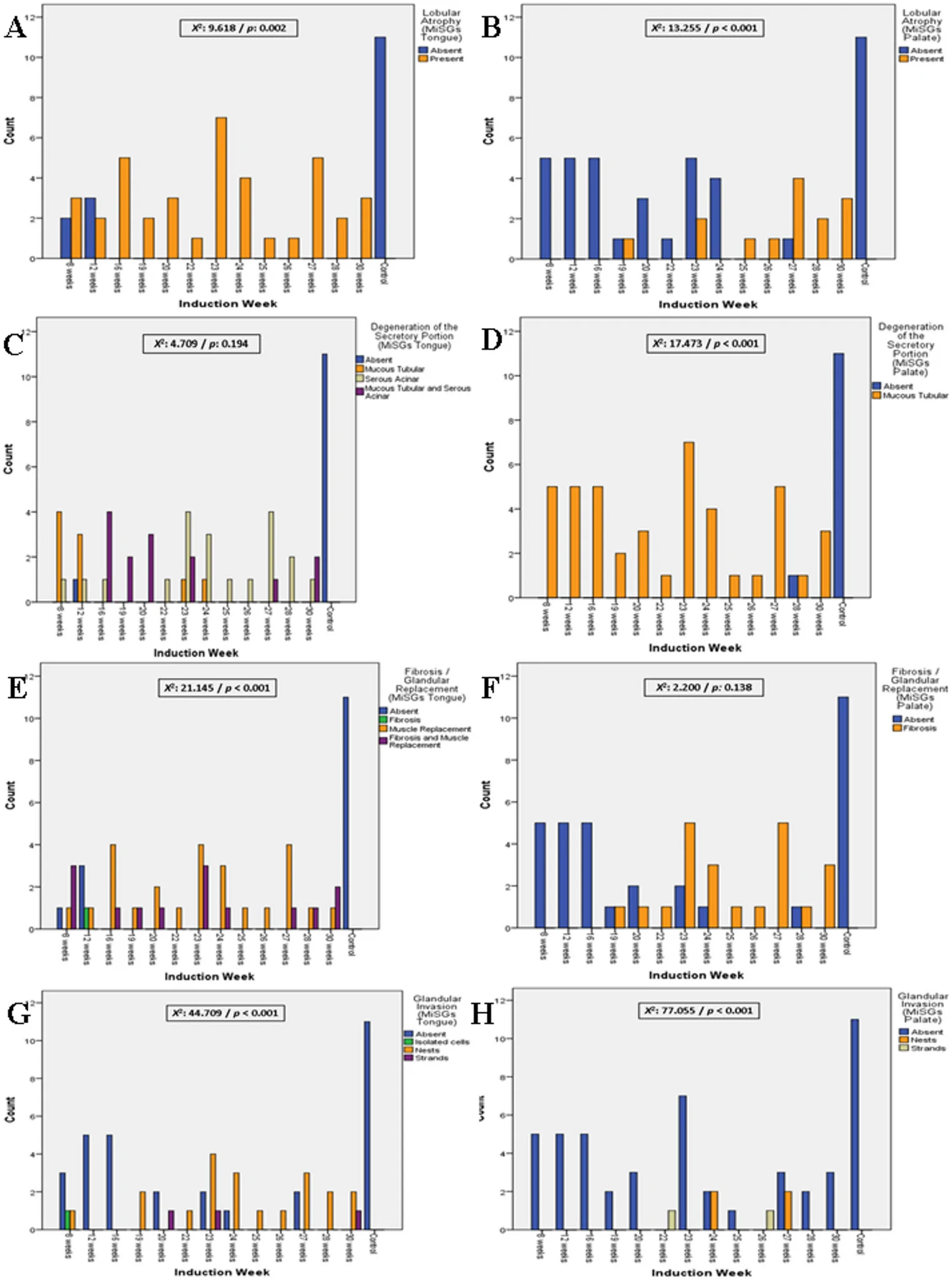
Bar charts showing the histopathological features observed in minor salivary glands (MiSGs): **A** Lobular atrophy in MiSGs of the tongue; **B** Lobular atrophy in MiSGs of the palate; **C** Degeneration of the secretory portion in MiSGs of the tongue; **D** Degeneration of the secretory portion in palatal MiSGs; **E** Fibrosis/Glandular replacement in tongue MiSGs; **F** Fibrosis/Glandular replacement in palatal MiSGs; **G** Glandular invasion in tongue MiSGs; **H** Glandular invasion in palatal MiSGs;

Lobular atrophy (Fig. 6A, B) of the MiSGs was most evident in the tongue, at 88.6% (*n* = 39), whereas in the palate it was observed in 14 cases (31.8%). Regarding degeneration of the secretory portion in the tongue (Fig. 6C), this change was present in 43 cases (97.7%), with 20 cases in serous acini (45.4%), 14 cases concurrently in mucous tubules (31.8%) and serous acini, and 9 cases (20.4%) only in mucous tubules. In MiSGs of the palate that present only a mucous tubular secretory portion (Fig. 6D), we observed this change in the same number of cases (n: 43). Regarding the presence of fibrosis or glandular replacement by other tissue, we found that 40 cases (91%) of lingual MiSGs (Fig. 6E) exhibited this change, with the destroyed portion of the gland replaced by muscle in 25 cases, 14 cases of concomitant fibrosis and muscular replacement, and 1 case of fibrosis alone. In palatal MiSGs, fibrosis (Fig. 6F) was present in 22 cases (50%). Invasion of tongue MiSGs (Fig. 6G) by squamous cell carcinoma was observed in 24 cases (54.5%), with the most common morphological pattern of invasion being nests (n: 20), followed by cellular strands in 3 cases and 1 case of invasion by isolated cells. In palatal MiSGs (Fig. 6H), invasion was present in only 6 cases (13.6%), with 4 cases showing a nests pattern and 2 cases showing cellular strands.

We found negative and statistically significant correlations (*r*_*s*_) between the week of experimental induction in tongue MiSGs and ductal hyperplasia (*r*_*s*_: − 0.305; *p*:0.024), ductal dysplasia (*r*_*s*_: − 0.358; *p*:0.007); lobular atrophy (*r*_*s*_: − 0.335; *p*:0.012); degeneration of the secretory portion (*r*_*s*_: − 0.343; *p*: 0.010); fibrosis/glandular replacement (*r*_*s*_: − 0.336; *p*:0.012). Negative and significant correlations in palatal MiSGs were also observed between the week of experimental induction and accumulation of saliva (*r*_*s*_: − 0.458; *p* < 0.001) and degeneration of the secretory portion (*r*_*s*_: − 0.711; *p* < 0.001).

A positive and statistically significant correlation was found between tongue MiSGs ductal dysplasia and ductal hyperplasia (*r*_*s*_: 0.485; *p* < 0.001); ductal metaplasia (*r*_*s*_: 0.408; *p*: 0.002); inflammatory infiltrate (*r*_*s*_: 0.424; *p*: 0.001); lobular atrophy (*r*_*s*_: 0.381; *p*: 0.004); degeneration of the secretory portion (*r*_*s*_: 0.442; *p*: 0.001); fibrosis/glandular replacement (*r*_*s*_: 0.351; *p*: 0.009). This same positive and statistically significant correlation was also observed between palatal MiSGs and accumulation of saliva (*r*_*s*_: 0.470; *p <* 0.001); inflammatory infiltrate (*r*_*s*_: 0.275; *p*: 0.042); degeneration of the secretory portion (*r*_*s*_: 0.291; *p*: 0.031).

Other positive and statistically significant correlations were also found between tongue MiSGs inflamatory infiltrate and ductal metaplasia (*r*_*s*_: 0.397; *p*:0.004); accumulation of saliva (*r*_*s*_: 0.463; *p <* 0.001); lobular atrophy (*r*_*s*_:0.687; *p <* 0.001); degeneration of the secretory portion (*r*_*s*_: 0.568; *p <* 0.001); fibrosis/glandular replacement (*r*_*s*_: 0.549; *p <* 0.001) and glandular invasion (*r*_*s*_: 0.694; *p <* 0.001). This same positive and statistically significant correlation was also observed between palatal MiSGs inflamatory infiltrate and ductal metaplasia (*r*_*s*_: 0.439; *p*:0.001); accumulation of saliva (*r*_*s*_: 0.292; *p*: 0.031); lobular atrophy (*r*_*s*_: 0.508; *p <* 0.001); degeneration of the secretory portion (*r*_*s*_: 0.436; *p*:0.001); fibrosis/glandular replacement (*r*_*s*_: 0.533; *p <* 0.001) and glandular invasion (*r*_*s*_: 0.277; *p*:0.041).

Regarding the relationship between the presence of changes in the evaluated MiSGs and the histopathological diagnosis in the superficial epithelial component, we observed glandular changes in cases of epithelial dysplasia and carcinoma in situ; however, the greatest number of changes in MiSGs were associated with OSCCs.

**Fig. 7:**
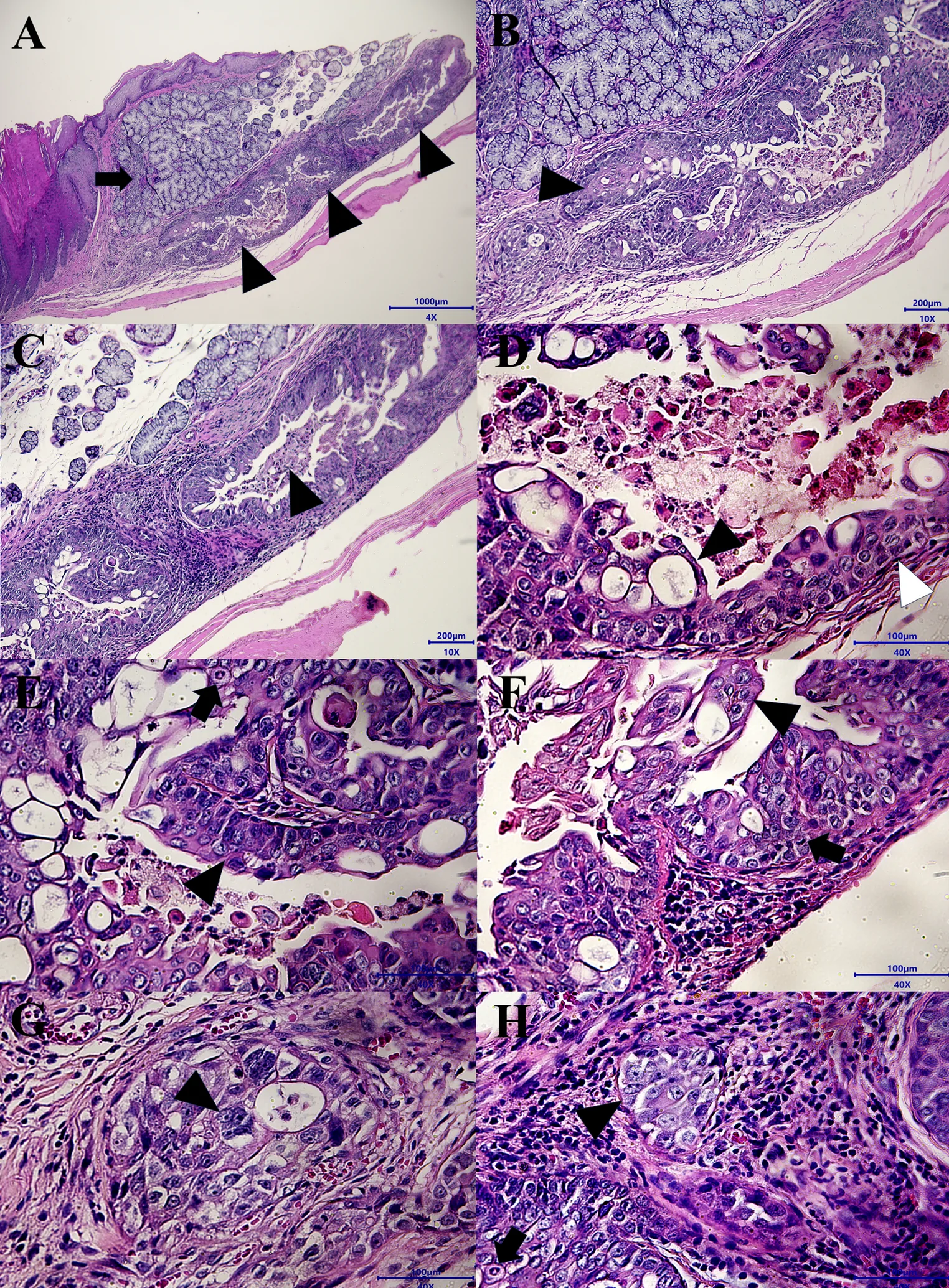
**A** Presence of ductal carcinoma in a minor salivary gland of the palate (arrowhead) and an area of solid invasion (arrow) of the gland (HE, 4x objective); **B** Area of squamous differentiation (arrowhead) in the ductal carcinoma (HE, 10x objective); **C** Comedonecrosis (arrowhead) in the lumen of the ductal carcinoma (HE, 10x objective); **D** Cribriform pattern with a “Roman Bridge” appearance (black arrowhead), presence of atypical mitotic figures (black arrow) and flattened myoepithelial cells (white arrowhead) (HE, 40x objective); **E** Papillary pattern within the lumen showing nuclear and cellular pleomorphism (arrowhead), as well as atypical mitotic figures (arrow) (HE, 40x objective); **F** Papillary projection (arrowhead) and typical mitotic figure (arrow) (HE, 40x objective); **G** Area of solid invasion (arrowhead) showing vesicular nuclei, rounded nucleoli, and the presence of a lumen (HE, 40x objective); **H** Solid pattern of invasion (arrowhead) and atypical mitotic figure (arrow) (HE, 40x objective)

**Fig. 8:**
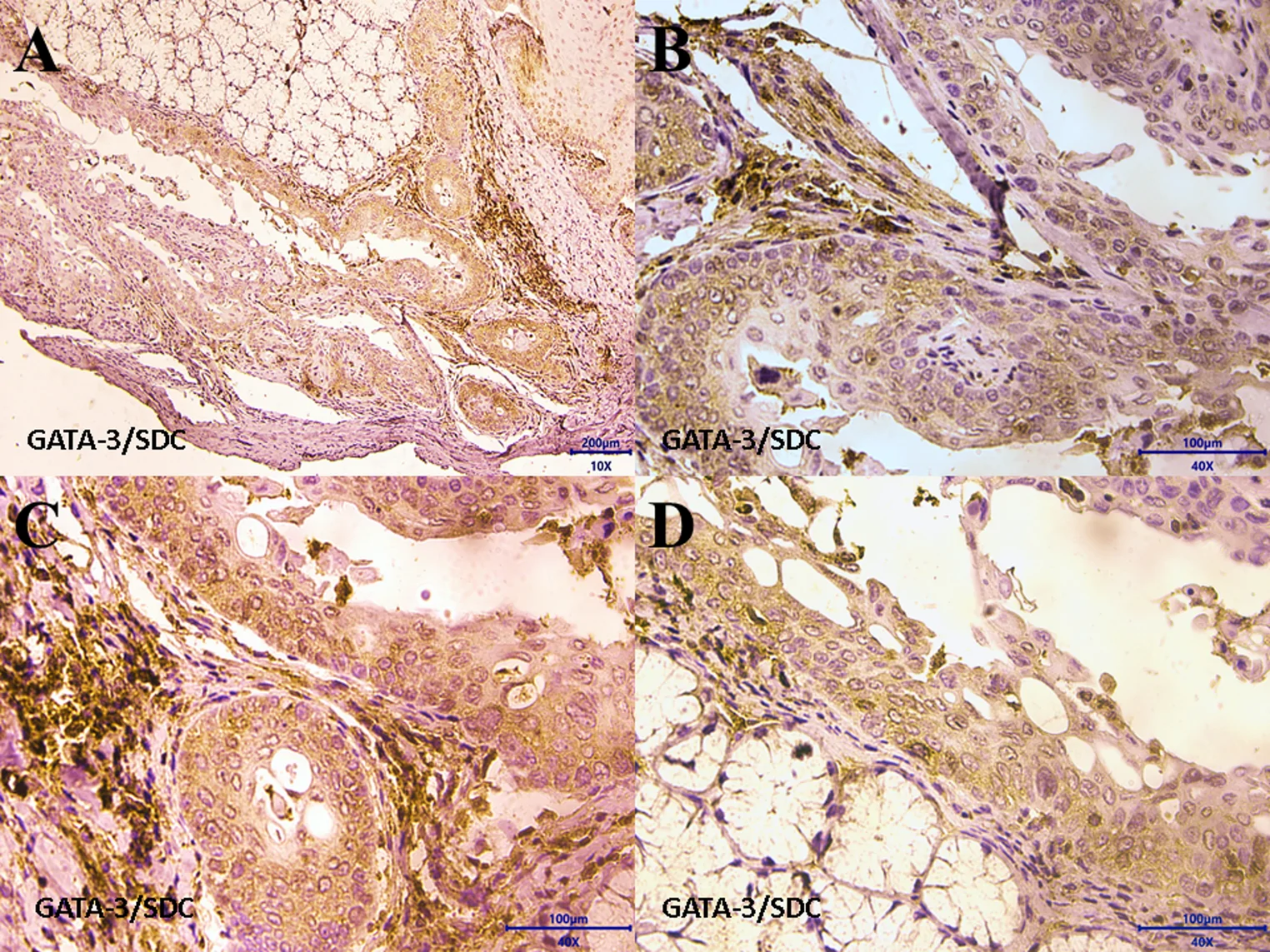
**A** Photomicrograph of salivary duct carcinoma showing immunopositivity for Gata-3 (4x objective/HiDef); **B** Positive immunoreactivity in the lining cells and inflammatory cells present in the stroma of salivary duct carcinoma (40x objective/HiDef); **C** Evident immunoreactivity in neoplastic islands of salivary duct carcinoma (40x objective/HiDef); **D** Immunostaining for Gata-3 in the epithelial component showing a cribriform pattern with a “Roman Bridge” appearance (40x objective/HiDef)

In our histopathological analysis, we observed the presence of an incidental salivary ductal carcinoma in the palate of a rat 22 weeks into the experimental induction of oral carcinogenesis. It was characterized by ducts lined by an epithelial component exhibiting three basic architectural patterns: cribriform, papillary, and solid. In the cribriform areas, we observed wide intercellular spaces resembling a Roman bridge. In the lumen, we observed the presence of necrotic material (comedonecrosis), the epithelial cells exhibited a predominantly eosinophilic cytoplasm, sometimes amphophilic, with nuclear atypia, vesicular nuclei, round nucleoli, and the presence of flattened myoepithelial cells. In addition to the presence of atypical mitotic figures and areas of perineural and perivascular invasion with a more solid pattern (Fig. 7A–H). The salivary duct carcinoma underwent immunohistochemical analysis, which revealed Gata-3 positivity in the cells of the parenchymal component and in predominantly inflammatory stromal cells, supporting the ductal differentiation (Fig. 8A–D).

## Discussion

As a method for developing oral carcinogenesis models for study, experimental chemical induction using 4NQO is the most widely used approach in animal models (Schuch et al. 2024). This model simulates how chronic tobacco use contributes to oral cancer (Sur et al. 2018). DNA is a target molecule for 4NQO and its metabolites, which form adducts within the genome, promoting mutagenesis and tumor formation. Oxidative stress is believed to play a pro-carcinogenic role in carcinomas caused by 4NQO (Ferreira de Aguiar et al. 2006). In the oral cavity, this chemical agent predominantly affects the surface of the epithelial component, causing the onset of epithelial dysplasia and carcinoma; these lesions may extend deeper into the ductal system, sometimes causing changes in the secretory portion of the minor salivary glands (Bhola et al. 2023).

An aspect not widely explored in research is the analysis of precursor changes present in the salivary glands that may lead to the development of neoplasms. Unfortunately, the difficulty in early detection of these changes is related to the absence of specific clinical or imaging findings, as well as consistent phenotypic alterations (Gunhan et al. 2019).

Accordingly, we performed histopathological analysis of the minor salivary glands of the tongue and palate in cases treated with 4NQO in the experimental groups from the 8th to the 30th week of oral carcinogenesis induction. We found that hyperplasia, metaplasia, and ductal dysplasia, as well as lobular atrophy, fibrosis, and glandular invasion, occurred predominantly in the lingual MiSGs. The same number of cases was found in the MiSGs of the tongue and palate when observing the accumulation of saliva within the duct with ductal ectasia, as well as degeneration of the secretory portion. Regarding the inflammatory infiltrate, palatal MiSGs were the most affected; however, there was a predominance of mild inflammatory infiltrate. In contrast, moderate to intense inflammatory infiltrate was more evident in lingual MiSGs. The evidence of greater involvement of lingual MiSGs in a model of carcinogenesis induction of the epithelial lining demonstrates that the action of 4NQO, which mimics tobacco, is not restricted to the epithelial surface and reinforces that certain alterations, such as ductal dysplasia, are evident. An important point to consider is that this higher percentage of abnormalities found in tongue MiSGs may be related to the highly irregular surface of the dorsal portion of the tongue, which keeps the carcinogen in contact with the lining epithelium for a longer period of time, as well as the presence of a greater number of excretory ducts in the tongue compared to the palate, increasing contact with the ductal epithelium and secretory regions.

In the study by Bhola et al. (2023), an analysis of the minor salivary glands in different regions of the oral cavity was conducted, revealing a higher number of cases presenting with ductal hyperplasia, inflammatory infiltrate, degeneration of the secretory portion, and glandular invasion. However, in our study, we found that the changes most commonly affecting the lingual and palatal salivary glands were: degeneration of the secretory portion, inflammatory infiltrate, accumulation of saliva within the duct, and fibrosis or other glandular replacements. It is important to clarify that changes such as hyperplasia and metaplasia are reversible adaptive responses to any stimulus; however, their occurrence and persistence are cause for concern, as they can create an environment conducive to dysplastic changes and malignant transformation. Changes in the secretory portion of the glands and fibrosis may also be related to adaptation; however, they may be attributed to ischemia caused by the carcinogen 4NQO.

Upon analyzing the secretory portion of the lingual MiSGs, we observed that degeneration of the secretory portion was most evident on the 8th and 12th days, with a decrease throughout the experiment, whereas the serous acinar glands showed an increase as the experimental weeks progressed. In contrast, in the palatal MiSGs, which consist solely of mucous tubules, considerable degeneration was observed from the 8th to the 16th week, peaking at the 23rd week and decreasing through the 30th week. In the study by Vered et al. (2003), the authors did not observe significant histopathological changes in the secretory portion of the minor salivary glands studied up to 28 weeks of experimentation. A possible explanation for this divergent finding may lie in the dilution used; in our study, we used double the concentration, which allowed us to identify this change as early as the 8th week of experimentation.

The protective role of saliva against the mechanism of carcinogenesis has been noted; experimental studies have shown that the onset of squamous cell carcinoma occurred earlier in animals without salivation, suggesting that this same process occurs in humans (Sioga et al. 2006; Kaplan et al. 2001). In the present study, we observed that the destruction of the secretory portion, primarily of the mucous tubular glands found in both the minor salivary glands of the tongue and the palate, will compromise one of the protective mechanisms against the action of carcinogens, given that the minor salivary glands are responsible for approximately 70% of mucus production in the oral cavity.

Mohan et al. (2016) report that hyperplasia and metaplasia are adaptive changes that, when occurring in the excretory duct, may be related to the effect of chemical carcinogens or associated with oral squamous cell carcinoma. Although these are reversible changes, their presence is undesirable, and their persistence may predispose to ductal dysplasia and subsequent malignant transformation. In the present study, we found that the presence of ductal hyperplasia and metaplasia was more frequent in MiSGs of the tongue, predisposing to the development of ductal dysplasia. Changes in the secretory portion of the glands and fibrosis may also be related to adaptation; however, they may be attributed to ischemia caused by the carcinogen 4NQO.

Another important finding of our study was the presence of ductal dysplasia, with a higher percentage observed in tongue MiSGs compared to the palate; this finding may be related to the more aggressive nature of squamous cell carcinoma of the tongue. Sarode et al. (2021) highlighted that the origin of oral squamous cell carcinoma is not restricted to the surface epithelium, but is also related to the transition zone between the surface epithelium of the oral mucosa and the excretory duct of the salivary glands. The action of carcinogens is plausible, potentially leading to dysplasia of the reserve cells present in the excretory duct, as well as compromise of the gland. In the face of evidence of a premalignant or malignant lesion in the surface epithelium, one should be concerned about possible ductal dysplasia in MiSGs.

Regarding the accumulation of saliva within the ducts and ectasia, which were observed with equal frequency in this study in both lingual and palatal MiSGs, we partially agree with the suggestion by Bhola et al. (2023) that links saliva accumulation to the presence of mucosal metaplasia. According to our study, a very close relationship was found in MiSGs between saliva accumulation within the ducts and ductal metaplasia, with very similar percentages. However, we found that saliva accumulation was significantly higher in cases of ductal metaplasia in palatal MiSGs. We believe that other factors may also contribute to this saliva retention, such as ductal hyperplasia.

Regarding glandular atrophy, we observed a very close relationship with the percentage of inflammatory infiltrate, around 86.4%, which was predominantly moderate to intense in MiSGs of the tongue. This relationship was not as evident in MiSGs of the palate; although 100% of cases presented inflammatory infiltrate, most cases were of mild intensity. This clearly indicates, in both cases, the presence of an immune response to the aggressive agent throughout the experimental weeks.

Upon analyzing the presence of fibrosis in minor salivary glands (MiSGs), we found that this replacement of the minor salivary gland by connective tissue was present in a higher percentage of cases involving the palate. In contrast, in cases of MSGs from the tongue, we found that the gland was replaced not only by fibrosis but also by muscle tissue or a combination of these two tissues. Vered et al. (2003) highlighted that glandular changes consist of extensive damage to the parenchyma, particularly in the secretory portion, with involution, glandular atrophy, and ultimately fibrosis, characterizing the irreversibility of the damage.

Regarding the invasion of MiSGs by squamous cell carcinoma, a stronger association was observed with tongue MiSGs, with a higher number of cases between the 19th and 30th weeks. A pattern of invasion was observed predominantly in the form of neoplastic nests, followed by strands and some cases of isolated cells. In contrast, in palatal MiSGs, invasion was much lower, occurring between the 22nd and 27th experimental weeks with a pattern of invasion predominantly in the form of nests, followed by strands.

One interesting finding was the detection of an incidental case of salivary ductal carcinoma (SDC) in palatal MiSGs in a rat 22 weeks after the induction of oral carcinogenesis. According to Luna-Ortiz et al. (2022), this is a rare malignant neoplasm, sometimes similar to ductal carcinoma of the breast, with an extremely poor prognosis. Dababneh et al. (2024) reported that SDC can arise de novo or from a preexisting salivary gland neoplasm, usually a pleomorphic adenoma. In the case of the salivary ductal carcinoma found in our study, we used 4NQO diluted in the rats’ drinking water to induce oral carcinogenesis, successfully obtaining OSCCs of the tongue and palate.

Nielson et al. (2023) note that the differential diagnosis of SDC includes both primary salivary gland tumors and metastatic carcinoma. High-grade mucoepidermoid carcinoma or high-grade transformation of any primary salivary gland tumors can pose a diagnostic challenge. In these cases, it is prudent to carefully search for areas of lower grade and areas with the classic histology of these primary tumors. In addition, metastatic squamous cell carcinoma must be ruled out; immunohistochemistry for p63/p40 and CK5/6 will be positive in SCC, whereas AR and Gata-3 should help identify ductal differentiation. Udager and Chiosea (2017) add that oncocytic carcinoma and cystadenocarcinomas not otherwise specified may be part of the differential diagnosis of SDC. Oncocytic carcinoma can be distinguished from SDC by its more granular cytoplasm and higher mitochondrial content, observable at the ultrastructural level or with hematoxylin and phosphotungstic acid staining. Furthermore, oncocytic carcinoma and cystadenocarcinoma do not exhibit prominent comedonecrosis or apocrine differentiation.

In our study, we observed changes in the ductal system, parenchyma, and glandular stroma of the MiSGs; we identified the histological features characteristic of SDC in one case involving MiSGs of the palate; and immunoreactivity for Gata-3 supports the ductal differentiation of this lesion. However, to date, there are no reports in the literature regarding the possible induction of ductal carcinomas through the oral administration of 4NQO. Important to note that this is a preliminary observation that requires further study.

Nielson et al. (2023) reported the aggressive behavior of this neoplasm, which exhibits high-grade cytology, comedonecrosis, and cribriform architecture. It may present with invasive or in situ components, with rare reports of purely in situ SDC. The intraductal components are identified by their characteristic architecture, including the cribriform pattern with a “Roman bridge” appearance, as well as papillary and solid patterns exhibiting cellular and nuclear pleomorphism, along with the presence of mitotic figures. Regarding the invasive component, we observe the presence of nests, strands, and solitary cells, with perineural and lymphovascular invasion being common. In our case diagnosed as salivary ductal carcinoma of the palate, we identified the typical histopathological features described above, as well as the presence of perineural and perivascular invasion.

## Conclusion

The present study demonstrated the presence of changes in salivary glands of the tongue and palate in all cases of rats subjected to chemically induced oral carcinogenesis; the changes described were frequently associated with oral squamous cell carcinomas (OSCCs), carcinomas in situ, and oral epithelial dysplasias. The most evident alteration in both tongue and palate MiSGs was degeneration of the secretory portion, reducing salivation and promoting the oncogenic action of 4NQO. However, alterations such as hyperplasia, metaplasia, and dysplasia, which may promote the development of neoplasms, were more prevalent in tongue MiSGs. Given this, it is important for the professional to be attentive to possible changes in MiSGs when there is a diagnosis of a potentially malignant or malignant lesion in the superficial epithelial component, because these changes present in MiSGs may be related to the development of other malignancies.

## Data Availability

No datasets were generated or analysed during the current study.
